# A teachable moment communication process for smoking cessation talk: description of a group randomized clinician-focused intervention

**DOI:** 10.1186/1472-6963-12-109

**Published:** 2012-05-03

**Authors:** Susan A Flocke, Elizabeth Antognoli, Mary M Step, Sybil Marsh, Theodore Parran, Mary Jane Mason

**Affiliations:** 1 Department of Family Medicine, Case Western Reserve University, Cleveland, OH, USA; 2 Department of Epidemiology and Biostatistics, Case Western Reserve University, Cleveland, OH, USA; 3 Case Comprehensive Cancer Center, Cleveland, OH, USA; 4 Prevention Research Center for Healthy Neighborhoods, Case Western Reserve University, Cleveland, OH, USA; 5 Division of Medicine, MetroHealth Medical Center, Cleveland, OH, USA; 6 Department of Medicine, Case Western Reserve University, Cleveland, OH, USA; 7 Department of Family Medicine, 11000 Cedar Ave, Suite 402, Cleveland, Ohio, 44106-7136, USA

**Keywords:** Smoking cessation, Health behavior change, Doctor-patient communication, Primary care, Study protocol, Teachable moments

## Abstract

**Background:**

Effective clinician-patient communication about health behavior change is one of the most important and most overlooked strategies to promote health and prevent disease. Existing guidelines for specific health behavior counseling have been created and promulgated, but not successfully adopted in primary care practice. Building on work focused on creating effective clinician strategies for prompting health behavior change in the primary care setting, we developed an intervention intended to enhance clinician communication skills to create and act on teachable moments for smoking cessation. In this manuscript, we describe the development and implementation of the Teachable Moment Communication Process (TMCP) intervention and the baseline characteristics of a group randomized trial designed to evaluate its effectiveness.

**Methods/Design:**

This group randomized trial includes thirty-one community-based primary care clinicians practicing in Northeast Ohio and 840 of their adult patients. Clinicians were randomly assigned to receive either the Teachable Moments Communication Process (TMCP) intervention for smoking cessation, or the delayed intervention. The TMCP intervention consisted of two, 3-hour educational training sessions including didactic presentation, skill demonstration through video examples, skills practices with standardized patients, and feedback from peers and the trainers. For each clinician enrolled, 12 patients were recruited for two time points. Pre- and post-intervention data from the clinicians, patients and audio-recorded clinician‒patient interactions were collected. At baseline, the two groups of clinicians and their patients were similar with regard to all demographic and practice characteristics examined. Both physician and patient recruitment goals were met, and retention was 96% and 94% respectively.

**Discussion:**

Findings support the feasibility of training clinicians to use the Teachable Moments Communication Process. The next steps are to assess how well clinicians employ these skills within their practices and to assess the effect on patient outcomes.

**Trial Registration:**

ClinicalTrials.gov Identifier: NCT01575886

## Background

Effective clinician-patient communication about health behavior change is one of the most important and overlooked strategies to promote health and prevent disease [1-3]. Modifiable behavioral risks such as tobacco use, lack of exercise, and poor diet choices contribute to more than 50% of preventable mortality and morbidity in the United States [4-7].Nationally, 87% of adults visiting a primary care clinician are overweight, smoke cigarettes or fail to exercise at the recommended levels [8]. Most Americans see a primary care clinician at least once during the year [9], providing a unique opportunity to reach the majority of the population with health behavior change messages that are tailored by knowledge of medical history and a continuity relationship. While guidelines for specific health behavior counseling have been created and promulgated [10-13],evidence of the successful adoption of such research findings in actual primary care settings is often lacking [14,15].Potential barriers to implementation include concerns about competing demands for time [16-19], anticipated patient resistance to cessation counseling [20-22],and the lack of effective strategies for accomplishing the recommendations within the routine flow of patient care.

Our ongoing research is generating rich evidence about clinicians’ natural use of ‘teachable moments’ for addressing health behavior change during the primary care outpatient visit [23,24]. A teachable moment involves linking a health behavior to a salient patient problem and creating an opportunity for effective health behavior change discussions [25]. While clinicians commonly use teachable moments to *initiate* health behavior talk, communication skills for navigating patient resistance to behavior change are generally lacking, resulting in inefficient and ineffective use of time. This deficiency in communication skills may be overcome by pragmatically adapting aspects of behavioral counseling to the primary care setting. For example, brief motivational interviewing and patient-centered therapy techniques promote specific communication skills for eliciting the patient’s perspective, adapting to resistance, and partnering to encourage behavior change [26-28].These strategic communication skills complement the teachable moment, and create a context conducive to health behavior change.

Building on this work and partnering with experts in medical education and communication, we developed a communication process that teaches clinicians to capitalize on teachable moments by providing them with specific communication techniques that can be realistically translated into busy primary care practices with potentially powerful effects. We designed a group randomized trial to evaluate the effectiveness of this clinician-focused intervention. In this manuscript, we describe the overall research design, the development and design of the Teachable Moment Communication Process (TMCP) intervention, and the baseline characteristics of the participating clinicians and patients. We also report participant feedback regarding the clarity and utility of the intervention.

## Methods/Design

### Study aim

The overall aim of the clinical trial is to implement and evaluate a clinician‒focused intervention designed to facilitate health behavior change discussion. The two study questions are: 1) Does the TMCP intervention increase clinician use of the teachable moment communication process in the clinician's own practice?, and 2) Does use of the TMCP improve immediate and intermediate patient report outcomes related to smoking?

### Study design

**Figure 1:**
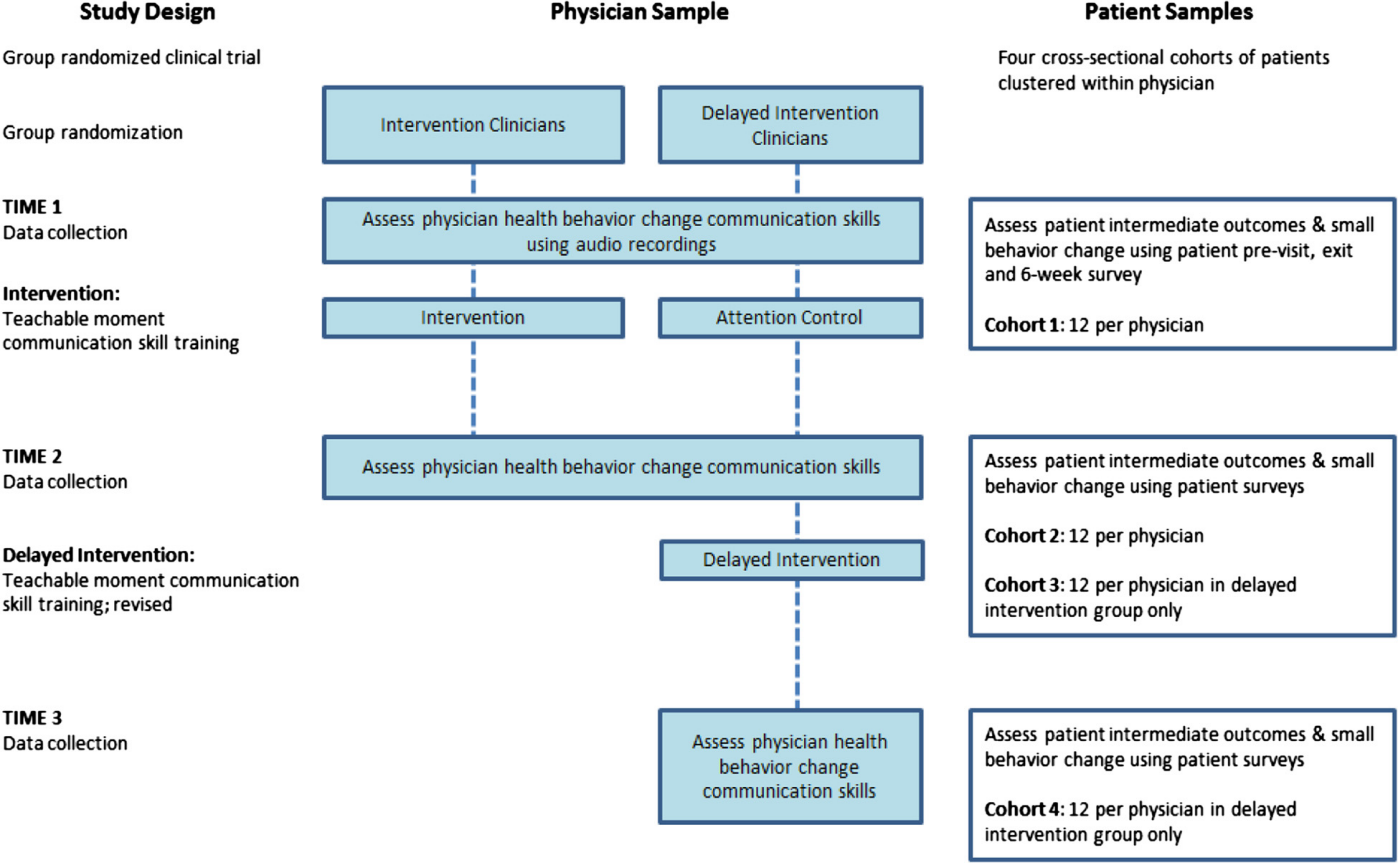
Study design and physician and patient samples.

The study design is a group randomized trial of 31 clinicians. Clinicians were randomized to either the intervention group or a delayed intervention group. Clinicians randomized to the delayed intervention group receive an attention control consisting of a multimedia education resource for colon cancer screening. After evaluation at the post-intervention data collection period, the delayed intervention group will cross over and receive a revised TMCP intervention (see Figure 1). The University Hospitals, MetroHealth Medical Center, and Cleveland Clinic Institutional Review Boards approved the study procedures.

### Clinician recruitment

**Figure 2:**
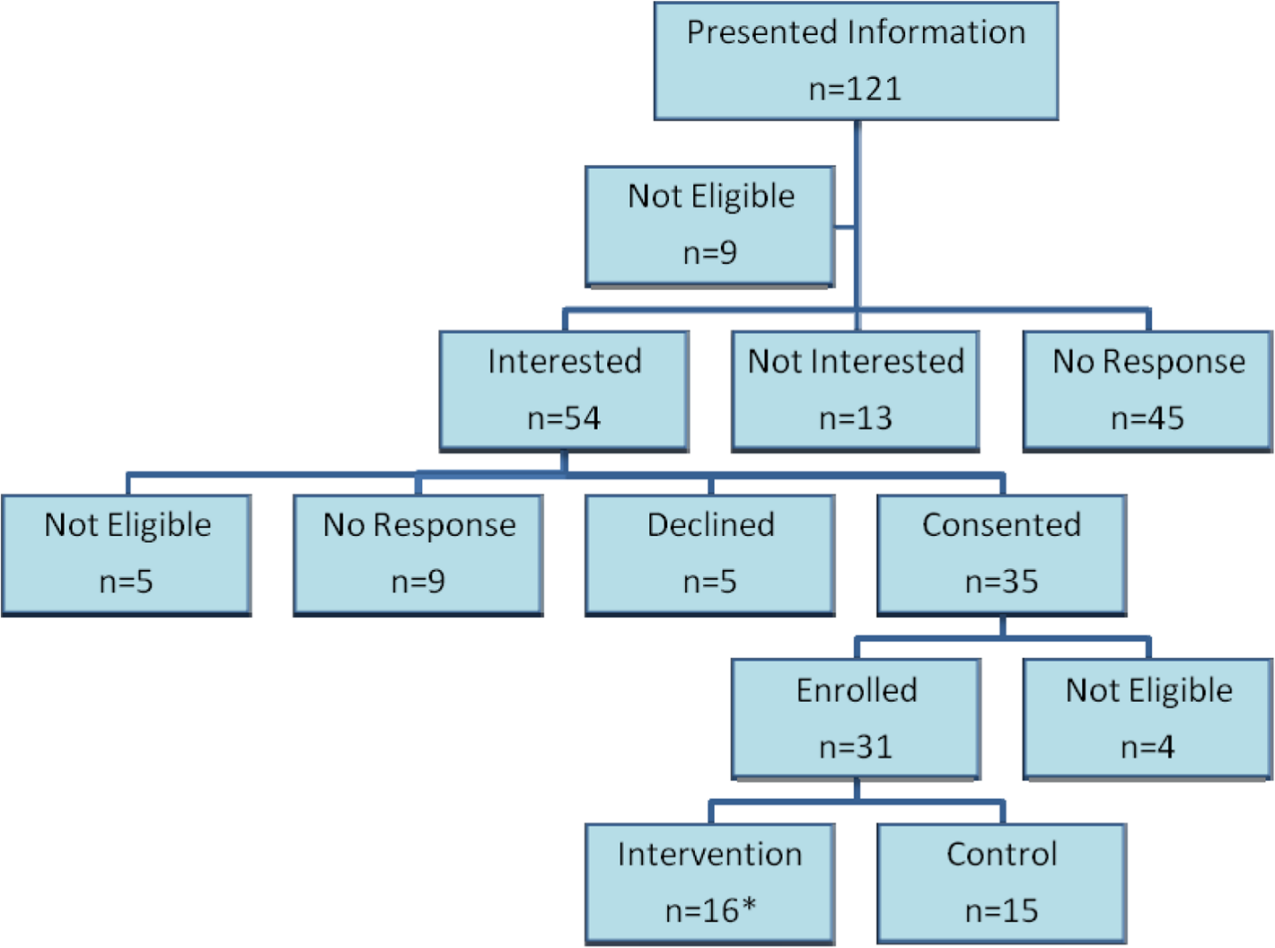
Physician recruitment for study participation.

The Research Association of Practices (RAP), a practice-based research network, and community satellite practices affiliated with one of the three main health care systems in the area, were the starting points for recruiting clinician participants. Clinicians were required to be in a community practice (i.e. not a residency practice or a practice located within the hospital), have a minimum of 2 patient care days per week, see predominately adult patients, and be located within 25 miles of the research center. The study was introduced with email invitations and in-person group presentations about the project. All clinicians were asked to complete a screening form, which included demographics, practice characteristics, contact information and level of interest in the study. One on one meetings were conducted with interested clinicians to provide further details about participation, address questions, and, if interested, complete the informed consent process. Consented clinicians were asked if they had any colleagues who might be interested in participating, and any referred clinicians were contacted directly by phone or email about the study. Two additional exclusion criteria were added after the recruitment process began: a) clinicians who reported plans to leave practice in the next year, and b) reported average patient waiting times of less than five minutes. Figure 2 shows recruitment frequencies for clinicians approached for participation in the study.

The project coordinator worked with the clinician and his/her staff to schedule all data collection sessions and intervention training session dates. Clinician participants were told the study was about communication and health behavior change, but were blinded to the specific health behavior advice hypotheses. After beginning data collection, low accrual of patients for some clinicians made it infeasible to complete data collection within the study time frame. If the study team was not able to successfully recruit one patient after 20 hours of data collection effort at a clinician's practice, that clinician was ineligible for continued participation in the study. Four clinicians were excluded because of low volume of eligible patients.

### Randomization

The unit of randomization for this study was the clinician. Clinicians were randomized to the intervention and delayed intervention groups using covariate adaptive randomization [29]. Covariate adaptive randomization increases power and validity through selected covariate and sample size balance. New participants were assigned to the intervention group based on two specific covariates; sex and practice system, as well as previous assignments of participants already randomized. In the event that clinicians were from the same practice and practiced closely together (e.g. shared patient panels, husband and wife practicing together) they were randomized together. This decision was to reduce the chance of cross contamination through exposure to the intervention content by clinicians in the same practice but randomized into different intervention arms. Clinicians were enrolled on a continuous basis over the start-up period. Those clinicians determined to be ineligible after randomization were replaced by the next clinician enrolled in the study with the same covariate profile. This occurred for two cases.

### Patient recruitment

For each clinician enrolled, approximately 12 patients were recruited per cohort in order to provide adequate power to conduct the planned analyses and detect moderate sized differences between groups. Patients were approached in the waiting room and asked to complete a brief screening survey while waiting to see the enrolled clinician. For all patient cohorts, patient participation was limited to those aged 18–70 who spoke English or Spanish and visited a participating primary care clinician for care during the observation periods. Patients who reported currently smoking cigarettes or small cigars ‘some days’ or ‘every day’ and reported smoking, on average, at least one cigarette per day or one small cigar per week were eligible for participation.

**Figure 3:**
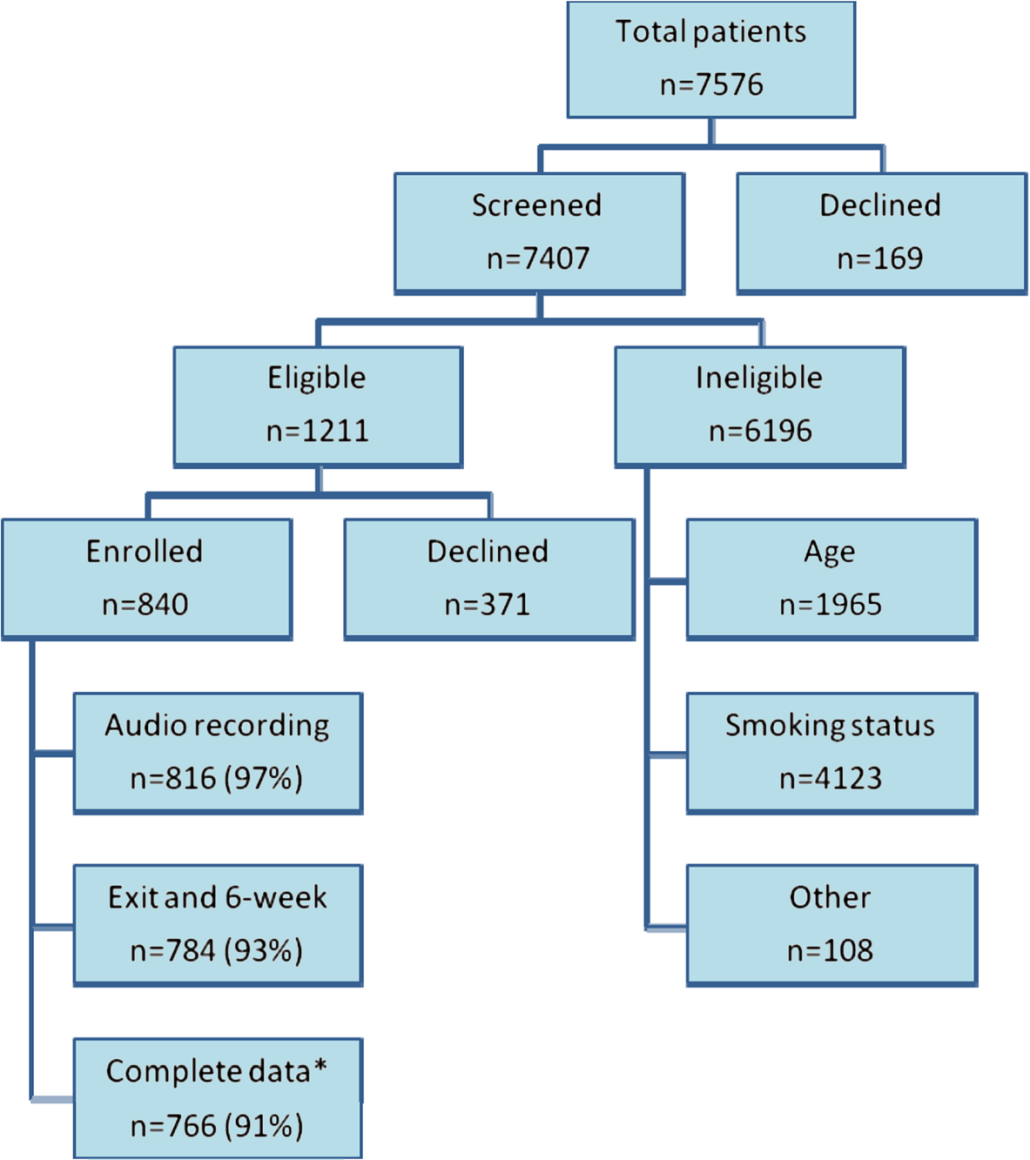
Patient recruitment for Cohort 1 and Cohort 2 combined.

After screening, eligible patients were invited to participate in the study. Patients were told that the study was about communication and may contain questions about health behaviors such as smoking, diet and exercise; they were not informed of any specific study hypotheses. Individuals who agreed to participate were consented in the privacy of the exam room before their visit. Patients were not eligible to participate twice in the same cohort and were excluded if they could not be reached by phone or mail for follow-up. Figure 3 shows recruitment frequencies for patients approached for participation in the study.

### Data collection

Data from the clinicians, patients and audio-recorded clinician‒patient interactions were collected. Data collection time points represent four cross‒sectional cohorts of patients. Each patient data collection observation included: 1) a pre‒visit patient survey administered by the data collector before the visit, 2) an audio recording of the patient visit, 3) a brief exit survey administered by telephone within 48 hours after the visit, and 4) a six‒week follow up survey administered by telephone. A total of 12 six-week surveys were completed by mail because we were unable to reach the participant by phone. A unique identifier, assigned to each patient at the time of screening, linked all surveys and the audio recording.

Measures assessed at each survey time point are presented in Table 1. Intermediate outcome measures include recall of smoking discussion, patient report of whether the smoking discussion was useful, patient perception of importance to quit and confidence they can quit, and patient-reported intention to quit. Patients were asked to rate importance and confidence of quitting smoking on a scale of 1-10 [10]. The patient’s intention to quit, based on the Transtheoretical Model [30], was assessed with the question ‘Are you considering quitting smoking within…'and the following response categories were presented: the next month; the next 6 months; the next year; more than 1 year’. Through prior work, the study team developed a 15-item self-report measure of behaviors and cognitions toward smoking cessation. Items were scored to compute the Incremental Behavior Change for smoking cessation (IBC-S) score. Potential adverse outcomes measured were patient satisfaction with the visit [31], satisfaction with communication [32]and visit duration. During the exit survey, patients were asked a global satisfaction item from the 9-Item Visit Rating Form on a 5-point Likert rating scale from poor to excellent. Patient satisfaction with the communication was measured using the Communication Assessment Tool, a 15-item scale with good reliability [32]. The duration of the visit was determined from the duration of face-to-face time with the physician in the exam room, as heard on the audio recording. Smoking discussion and performance of key features of the TMCP were coded using the audio recordings.

**Table 1:** Measures assessed at each study time point

**Outcomes & Characteristics**	**Source of measure**	**Pre-visit Survey**	**Exit Survey**	**6-week Survey**
Patient characteristics		X		
Self-reported health status	Global health item [33],	X		X
Smoking status	Items from BRFSS	X		X
Incremental behavior change	Developed by study team	X		X
Intention to change	Based on Transtheoretical Model [34]	X	X	X
Importance & confidence to quit	Rated on a 10 point Likert scale [35]	X	X	X
Recall smoking discussion	Single item [36].		X	
Satisfaction with communication	Communication Assessment Tool [32]		X	
Satisfaction with visit	Global item, Visit Rating Form [31]		X	

### Development of the intervention content

The content of the intervention was based in large part on our previous findings about how clinicians and patients naturally create teachable moments for health behavior change. Previously analyzed content of 811 audio recorded patient visits to primary care physicians, showed teachable moments to have four critical features [25]. First, there is discussion of a salient patient concern. This concern could be a symptom, worry or life issue that is meaningful to the patient, raised by either the clinician or the patient. Second, while discussing the patient’s salient concern, a transition, or link, is made to a relevant health behavior that is portrayed as problematic. Third, there is talk designed to motivate the patient to change that health behavior. This is accomplished by suggesting that changing the health behavior will improve the patient’s salient concern. Fourth, the patient accepts the portrayal of the health behavior as a relevant problem and that the health behavior ought to change. Here, the patient exhibits uptake of the health behavior change talk and may express a commitment to change.

This work indicated that across health behavior change discussions (n = 548), a teachable moment was observed to occur in just 11% of cases [25]. Missed opportunities (31%) and ‘teachable moment attempts’ (17%) were much more common. Missed opportunities lacked one or both of two key elements: a) a link from a patient's salient concern to the relevant health behavior and b) talk designed to motivate the patient to change that health behavior. The difference between a teachable moment and a teachable moment attempt was more nuanced. Teachable moment attempts are defined as discussion where there is a transition from a patient's salient concern to a relevant health behavior and a clinician attempt to motivate behavior change, but there is no collaborative uptake of the health behavior change talk by the patient or any expression of a commitment to change. Importantly, this lack of uptake is expressed as patient resistance to change. This resistance from the patient can lead to shaming or blaming language by the clinician, a clinician-dominated lecture, monologue, or argument. Such scenarios are not only ineffective at changing behavior, but can damage the patient-clinician relationship. Thus, while a teachable moment holds potential as an effective tool for addressing behavior change, integration of a strategy for negotiating patient resistance is necessary.

**Figure 4:**
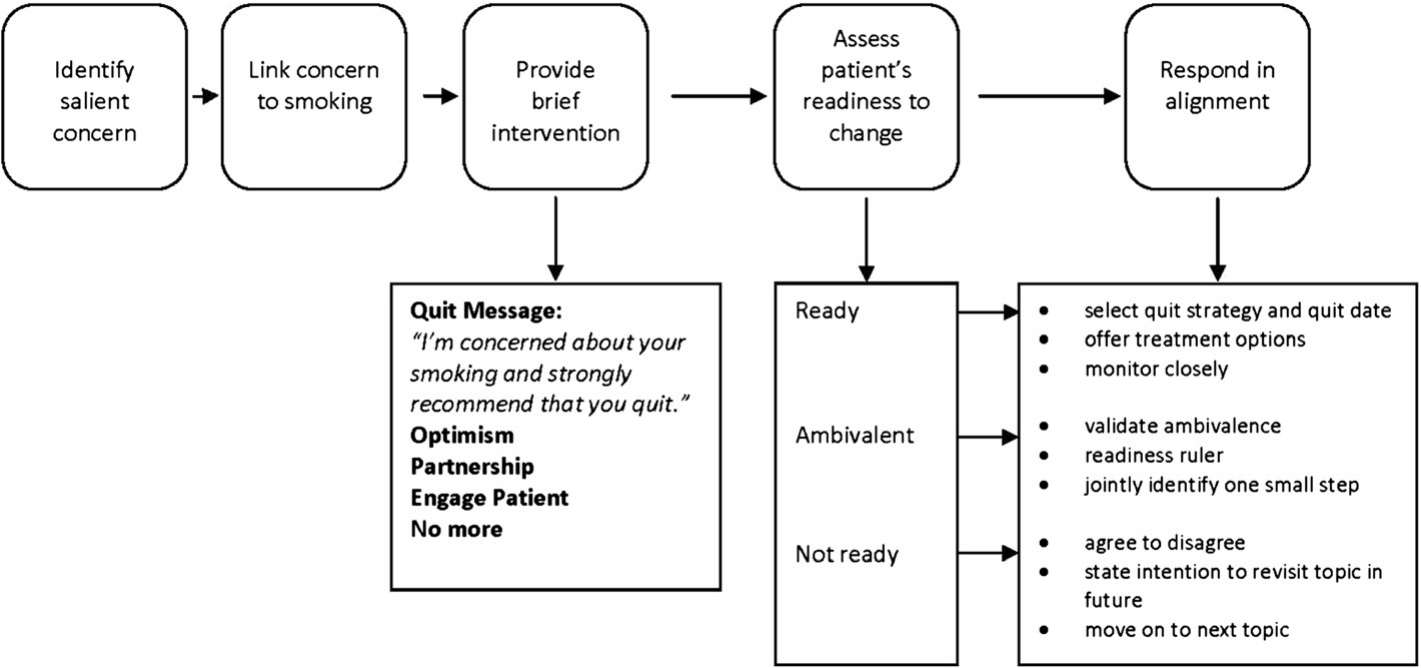
Schematic of the five elements of the Teachable Moment Communication Process.

Drawing on the frameworks and techniques of Motivational Interviewing, the Transtheoretical Model, and the principles of addiction medicine, we incorporated two fundamental communication skills into our new teachable moment model that address patient resistance: 1) an elicitation of the patient’s perspective about the proposed health behavior change and 2) a response by the clinician that is in alignment with the patient’s expressed level of readiness to change. Incorporating these elements into clinical interactions has the potential to make a major difference in the ensuing health behavior change discussion. The sequence of these communication tasks are represented in Figure 4 below. Moving step by step through the model, the Teachable Moment Communication Process begins with identification of a patient’s salient concern, and then links this concern to smoking behavior. Smoking is portrayed as germane to the patient’s salient concern and as problematic. Next, the clinician provides a brief quit message that conveys concern for the patient: 'I'm concerned about your smoking and strongly recommend that you quit'. This is followed by OPEN, a mnemonic representing Optimism, Partnership, Elicit, and No more (i.e., stop and listen to what the patient has to say). A similar approach has been used in medical education [37-39],where it was found to be an easy skill to learn with good face validity. This strategy is also congruent with maintaining a continuity relationship between clinician and patient. OPEN information is presented in a sentence or two that includes an expression of optimism that the patient is able to quit and offers the clinician’s partnership towards this end. Eliciting the patient’s perspective involves asking about the patient’s thoughts about the health behavior change and what actions might be next. For example, ‘My recommendation that you quit can be very hard to hear, so before we go any further, I am wondering what your thoughts are?’ Eliciting the patient’s perspective increases patient’s communication involvement [40] in the topic and almost always reveals the patient’s level of readiness for behavior change in the patient’s own words. Overall, the approach promotes clinician delivery of a clear, patient-centered behavior change message that encourages an accurate and honest assessment of the patient’s readiness to change, rather than acquiescence to what the patient thinks the clinician wants to hear. Furthermore, this strategy positions the clinician to respond in alignment with the patient’s expressed level of readiness.

Responding in alignment with the patient's expressed readiness to change increases the likelihood that the clinician’s response and proposed plan are acceptable to the patient, and reinforces a positive partnership. The goals of responding in alignment for someone who is ready to change included jointly identifying a quit strategy and a quit date, and monitoring very closely through phone calls or office visits. For the patient who is ambivalent about change, the goals are to validate the ambivalence that the patient feels about changing behavior and jointly identify one next small step. For the patient who is not ready to quit, the goal is simply to maintain a relationship that facilitates future discussion about smoking. The overall approach promotes a brief yet effective technique for discussing smoking cessation that both protects and takes strategic advantage of a positive clinician-patient relationship.

Starting with this approach and its specific components, we developed an intervention to teach clinicians the Teachable Moment Communication Process (TMCP) to address smoking cessation. The TMCP intervention teaches clinicians: (1) the skills necessary to recognize and foster teachable moments in clinical encounters, (2) strategies to effectively elicit the patients’ perspective on health behavior change, and express their alignment with that perspective, and (3) the ability to respond to the patient in a non-confrontational manner while providing brief advice appropriate to the patient’s expressed level of readiness to change.

### Intervention format, content, and fidelity

**Figure 5:**
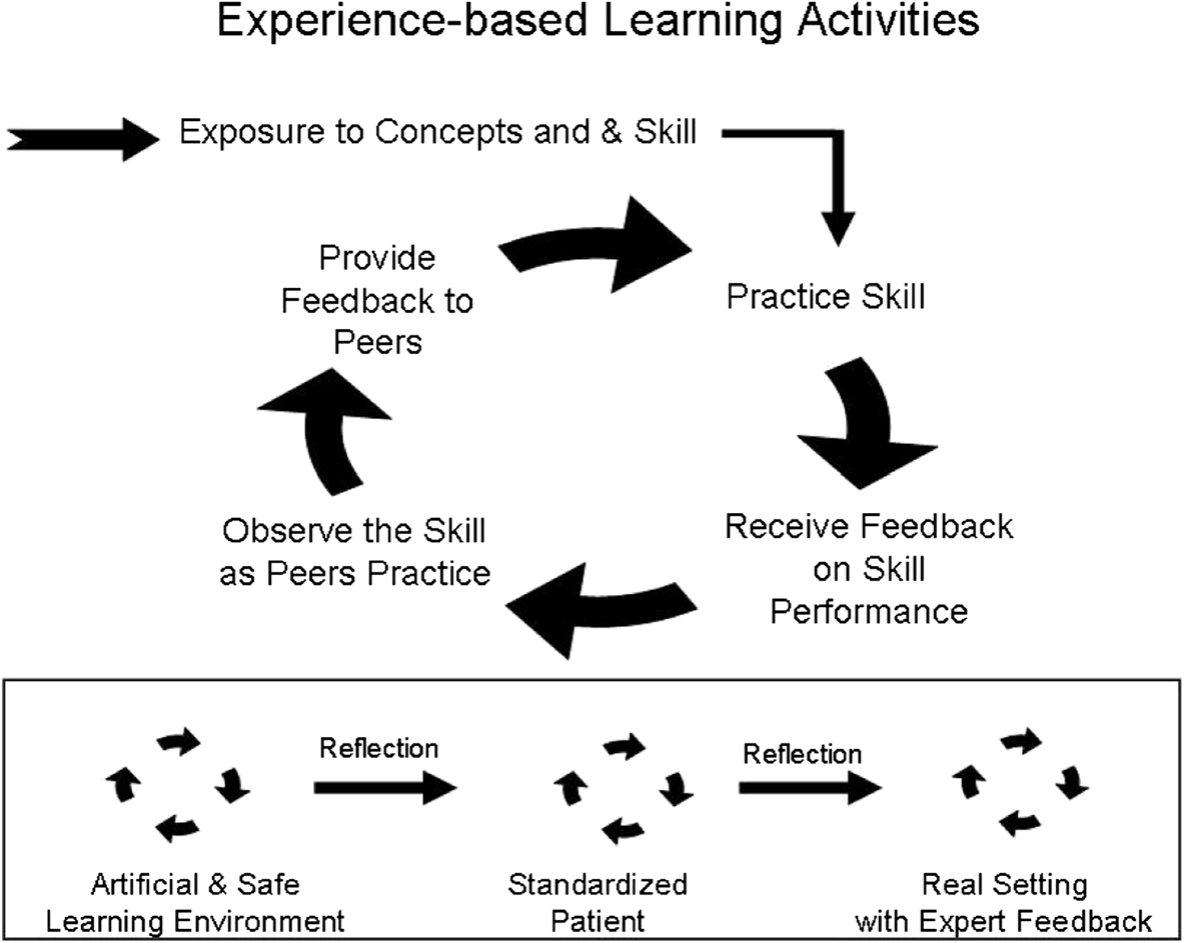
Cycle and progression of experience-based learning activities.

The intervention content was divided into two, 3-hour educational training sessions (Session 1 and Session 2). Sessions were designed to engage the learners with a mix of activities that included didactic presentation, skill demonstration and practice, and participant feedback during the skill practices. The training was guided by an experience-based learning approach focused on developing skills, receiving and providing feedback and moving from lower risk learning environments to real settings (see Figure 5). Participants worked in pairs for all but the didactic portions of the intervention and the final skills practice in session 2. Training sessions were held at the Mount Sinai Skills and Simulation Center (MSSSC), a state-of-the-art facility for health sciences education and evaluation. The MSSSC houses classrooms and simulated exam rooms equipped with video-recording capabilities that enabled video feedback of the communication skill practices.

**Figure 6:**
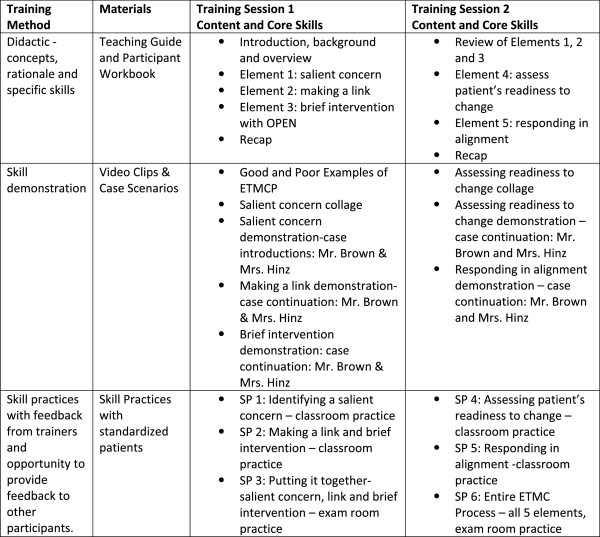
Method, materials and core skills for the intervention training sessions.

To ensure that all groups of participants received the same intervention, the format and content of the intervention were standardized by creating a teaching guide for presenters and corresponding workbook for participants. The teaching guide content was outlined and scripted so that each point delivered by the intervention trainers was replicable across groups. Participants were provided a workbook with content that paralleled the teaching guide, but that was oriented toward the participants’ point of view. Both sessions 1 and 2 began in the classroom setting where, after an introduction, participants were led through the five elements of the Teachable Moment Communication Process (see Figure 4). For each element, participants received: 1) a didactic presentation from one of the four training faculty, 2) a demonstration of that elements’ core skill via a video clip, 3) the opportunity to practice the core skill with a Standardized Patient (SP), and 4) the opportunity to both provide and receive feedback with a partner. The training content, core skills, and methods of training and evaluation are summarized in Figure 6.

#### Teaching guide and workbook

The teaching guide was created and edited over a period of six months in order to clearly and effectively communicate the content of the TMCP. The teaching guide featured scripted didactic portions for each trainer in order to ensure the content fidelity across all participant groups. This guide was broken into sections reflecting the 5-part Teachable Moment Communication Process. The trainers collaboratively developed the content of intervention and included: the project PI trained in health services research, a communication scientist, and two physicians with extensive training in addiction medicine. Each of the four trainers presented a section of the content. The participant workbook, developed in parallel with the teaching guide, provided a summary of each element, guides for putting each element into practice, an appendix listing local smoking cessation resources, and space for note-taking. These workbooks not only provided a guide for participants during their intervention skill practices, but also served as a reference for participants to take with them at completion of TMCP training. The teaching guide and workbook were paired via a slide presentation so that as the trainers moved through the intervention content, participants’ thinking was reinforced with simple, graphic visual content reflecting each TMCP element.

#### Video clips demonstrating skills

Examples of core TMCP skills were demonstrated using video clips interspersed through the didactic content. These clips followed two case examples through each TMCP element, illustrating specific communication skills used during an office visit. Additional clips were also produced to show multiple short examples that highlighted variability in the target concepts (e.g. salient concerns, or ways of expressing level of readiness to change). The training video was professionally produced and featured case portrayals by experienced standardized patients. The video content was used to further ensure replicability of the demonstrations across intervention groups.

#### Practicing skills with standardized patients ('skill practices')

Skill practices were included in the intervention as a way to learn behavioral enactment of that skill. Skill practices took place in the classroom immediately following each didactic and demonstration segment, and involved two participants and one SP working together. The SP had been trained to present a specific, realistic scenario to the participant that highlighted each TMCP component in order of presentation. The participant was provided information about the SP’s character, such as age, sex, and smoking history. Both participant and SP were instructed as to the objective of the reenactment and the stopping point. As participant A worked with the SP on a scenario, participant B observed, took notes in the workbook, and then provided feedback to participant A. A trainer was also assigned to each group to observe, keep the task was on track, and provide feedback to the participants. Participant then switched roles for a second scenario, thus having opportunity to practice each skill, and provide and receive feedback on his/her technique. Participants practiced consecutive component skills with the same partner, SP and patient scenarios. This familiarization with one partner and “patient” helped to provide continuity to the learning environment, so that each participant could more easily focus on the skill being taught.

Each training session culminated in skill practices that took place in simulated exam rooms using a series of different patient scenarios. In these skill practices (skill practices 3 and 6), participants: 1) worked with new standardized patients, 2) practiced skills in a more realistic exam room setting, 3) received feedback from trainers who observed through a one-way mirror, and 4) had their interactions video-recorded for later self-reflection and evaluation. The exam room skill practices combined each TMCP element that had been taught up to that point, and thus allowed participants to practice putting the elements together in naturalistic conversation. Exam room skill practices were also progressive in the sense that participants remained paired with their classroom partners, and following each SP encounter, shared feedback with their partner and a trainer. Training faculty who observed the interaction used a checklist of TMCP skills as a guide for providing additional insight. Then participants switched places and repeated this process before moving together to the next exam room and SP. In the final skill practice, participants enacted consecutive components of the entire TMCP, working individually and rotating through eight SP’s and eight new scenarios.

Using this method, participants were able to practice skills, observe others, and receive feedback from fellow clinicians and all of the trainers. Finally, these interactions were recorded and participants were instructed to observe their recorded interactions before their second session. This offered an opportunity for self-evaluation and self-reflection on the development of their new skills.

Session 3 of the intervention consisted of one of the intervention trainers meeting one-on-one with each participant. The trainer reviewed intervention team notes on the participant, and viewed the participants Skill Practice video clips in preparation for this session. This personalized session was scheduled approximately 1 week after the classroom sessions so that participants had an opportunity to use the skills in their own clinic. During the session, participants were debriefed about their experiences in Sessions 1 and 2, and provided feedback on their strengths and challenges in applying the TMCP, tips for refining their skills, and coaching on the challenges of skill implementation in the participants’ specific clinical context. The duration of this session was about 1 hour.

### Preparation to implement the intervention

Preparation of the final version of the TMCP intervention involved multiple phases of development and evaluation. Of importance for this report is the standardized patient training and the dress rehearsal and pilot test, which were used to finalize the content, choreography and timing of the training intervention.

#### Development of cases

Scenarios were based on actual primary care cases from a previous study conducted by the first author. Scenarios were designed to highlight a reason for the visit, a salient concern (which could be different from the main reason for the visit), and a level of readiness to change smoking. More than 25 cases were developed and 16 were ultimately selected and used for the intervention.

#### Standardized patients

Over the course of several months, the study team worked with staff at the MSSSC to recruit and train standardized patients (SPs), all of whom had prior SP experience. SP training involved an overview of the TMPC intervention objectives and format, and focused training on the concepts of a salient concern and levels of readiness to change. The bulk of instruction centered on enactment of patient scenario scripts where the SPs were required to convey a salient concern and a specific level of readiness to change. Scripts for the key elements of the cases were developed, read out loud, refined and then rehearsed using role play with a trainer playing the role of clinician as if in the skills practice sessions. Patient scenario scripts were brought to life through the SP's development of context and character, making the resulting simulated patient-clinician interaction more realistic.

#### Dress rehearsal

Due to the complex format and multiple learning modes employed in the TMCP intervention, a dress rehearsal was held to: 1) reveal potential unforeseen issues, 2) confirm the timing of each phase of the training session and of the intervention as a whole, and 3) provide realistic practice for the trainers and SPs. Study staff not involved in the development of the intervention sat in as participants. The dress rehearsal offered an important opportunity to choreograph movements between training spaces, and improve the clarity and wording of the didactic presentations. The dress rehearsal also provided an additional opportunity to train and refine the SP’s performance. Sessions 1 and 2 were rehearsed on separate days, and recorded for later review and discussion.

#### Pilot

After modifications based on the dress rehearsal were incorporated, the TMCP intervention was pilot tested in the MSSSC with a group of seven, second-year, Family Medicine residents from a community teaching hospital. The intervention content and procedures were delivered in the planned format and timing so that reactions to the content, performance of the skill practices, and transitions from one activity to the next could be observed and evaluated. Special attention was paid to how the residents transitioned through teaching modalities, and how effectively each resident-SP pair accomplished the specific communication skill required in each skill practice. After each session of the pilot, residents were asked to provide feedback by taking an on-line survey consisting of both close- and open-ended questions regarding the clarity, utility and applicability of the intervention content. Comments were reviewed and minor adjustments in the session were made based on pilot participant feedback as well as the intervention team's input on flow and timing.

### Data management & analysis

Survey data were entered into a Research Electronic Data Capture (REDCap) database hosted by Case Western Reserve University. REDCap is a secure, web-based application designed to support data entry, manipulation, reporting and export to statistical packages for research studies. Audio recordings were uploaded to a secure server to be coded qualitatively for elements of the TMCP. Upon completion of data collection, coding and survey data will be merged and analyzed using SAS statistical software v9.2 (Cary, NC). Survey and audio data were routinely monitored for completeness and quality over the course of data collection.

Data analyses reported in this manuscript include descriptive statistics of enrolled clinicians and patients in Cohorts 1 and 2. Demographic characteristics, visit characteristics and baseline smoking behavior were examined across all patients enrolled in the study. Characteristics of patients who declined participation were compared to those who enrolled in the study using Chi-square tests for categorical variables. Because the continuous variable number of cigarettes per day was not distributed normally, a Wilcoxen Rank Sum test was used to evaluate median differences between groups.

In future analyses of the overall study aims, the main independent variable will be the exposure to the TMCP intervention at the clinician level. All outcome variables are evaluated at the patient level and will be adjusted for the clustering of patients within clinicians using generalized linear mixed models (GLMM). First, we will examine clinician and patient characteristic differences at baseline (Cohort 1) between the TMCP intervention group (I) and the delayed intervention group (DI) using chi-square and t-tests. Differences with an effect size of 0.3 of a standard deviation for continuous variables, or 20% for categorical variables will be considered clinically significant and will be included in subsequent analyses as potential confounding variables.

The coded variables from the audio recording of the patient visits will be examined for Cohort 1. For this comparison, we expect that the I and DI groups will be similar in the use of the TMCP skills. The general model for these comparisons:

(1) $${\text{y}}_{\text{ij}}={\text{p}}_{0\text{j}}+{\text{p}}_{1\text{j}}{\left(\text{Patient characteristic A}\right)}_{\text{ij}}+{\text{p}}_{2\text{j}}{\left(\text{Visit characteristic b}\right)}_{\text{ij}}+{\text{e}}_{\text{ij}}$$

(2) $${\text{p}}_{0\text{j}}={\text{b}}_{00}+{\text{b}}_{01}\left(\text{TMCP Group}:\text{I}=1,\text{ DI}=0\right)+{\text{b}}_{02}\left(\text{Clinician characteristic C}\right)+{\text{r}}_{0\text{j}}$$

The Level 1, or patient visit level model represents the outcome (i.e. teachable moment communication process occurred) as a function of the clinicians mean at baseline plus a random error. Baseline patient or visit level confounding variables will be included in the model as covariates. The clinician level (Level 2) models specify the relationship between the clinician-level predictors and the coefficients in the Level 1 model, with a random effect. Additional clinician level covariates may be included at this level. β_00_ represents the mean outcome score across clinicians, TMCP group indicates the intervention condition and β_01_ represents difference in the outcome score that is attributed to the group assignment, controlling for other clinician and patient level variables in the model.

In this study design, the outcome measures are derived from the audio recordings and patient survey of a sample of patients at each time point. These data do not reflect repeated measures on the same patients across the time points, but rather separate cross-sectional cohorts. Thus, the model for the examination of Aim 1 will include a dummy variable at the patient level to indicate the cohort (Cohort 1 versus Cohort 2). We hypothesize that the odds of performing key elements of the TMCP during smoking cessation talk with patients will be twice as high for the intervention group as the delayed intervention group. For Aim 2, the attention will turn to the outcomes of patient recall and reported usefulness of smoking discussion, intention to quit, IBC-T change scores, and smoking status. Satisfaction with the communication and the duration of the visit in minutes will also be evaluated as potential adverse effects. The same generalized linear mixed model will be used with the indicator of TMCP group as the key independent variable.

## Results

Characteristics of participating clinicians randomized to intervention groups are displayed in Table 2. As evidence of satisfactory randomization, intervention and delayed intervention clinicians were similar in regard to all examined demographic information and practice characteristics.

**Table 2:** Characteristics of participating clinicians randomized to initial intervention and delayed intervention groups

	**Total** **(n = 31)**	**Delayed Intervention** **(n = 15)**	**Intervention** **(n = 16)**	***P***
Female (%)	48	47	50	0.85
Race, white (%)	87	87	88	0.95
Training (%)				
Internal Medicine	23	20	25	0.32
Family Medicine	71	80	63	
Nurse Practitioner	6	0	13	
Safety net practice (%)	65	67	63	0.81
Years since residency completed, mean	17.4	18.7	15.3	0.37
Patient care days per week, mean	4.1	4.0	4.1	0.75
Speaks Spanish during visits (%)	19	20	19	0.93

Sixty-nine percent of eligible patients agreed to participate in the study; their characteristics and the characteristics of eligible patients who declined participation are shown in Table 3. Patients who enrolled in the study were more likely to be female and African-American than those who declined. To further support randomization, patient participants seeing clinicians in the intervention and delayed intervention groups were not significantly different on the basis of any patient characteristics.

**Table 3:** Characteristics of patients that declined participation and enrolled in study

	**Declined** **(n = 371)**	**Enrolled** **(n = 840)**	***P***
Age category (%)			
18 to 29 years	18	16	0.37
30 to 39 years	19	18	
40 to 49 years	24	24	
50 to 59 years	24	29	
60 to 70 years	15	12	
Female (%)	54	61	0.01
Hispanic (%)	12	10	0.17
Race (%)			
White	64	56	<0.001
Black, African-American	23	34	
Other / more than one	12	7	
Education (%)			
Less than high school graduate	21	22	0.84
High school graduate or GED	37	35	
Some college	30	31	
College graduate	12	13	
Self-reported health status (%)			
Excellent	6	6	0.83
Very good	15	15	
Good	32	33	
Fair	31	30	
Poor	15	17	
Chronic conditions (%)			
None	51	49	0.12
One	28	25	
Two or more	20	26	
Seeing regular doctor (%)			
First visit	28	23	0.26
Known less than 1 year	14	19	
Known more than 1 year	58	59	
Reason for visit (%)			
New illness or problem	31	27	0.38
Continued care	44	48	
Well care, physical	25	25	
No. cigarettes smoked / day, median (IQR)	10 (5–20)	10 (5–20)	0.99
Considering quitting within the (%)			
Next month	----	28	
Next six months	----	27	
Six months to one year	----	22	
Not within the next year	----	23	
Importance of quitting, mean (SD)	----	7.9 (2.6)	
Confidence in quitting, mean (SD)	----	6.5 (2.9)	

The overall sample provides adequate power to conduct the planned analyses and detect moderate differences between intervention and delayed intervention groups. Power analyses prior to recruitment indicated a minimum sample of 28 physicians with 12 patients per cohort, with 10% loss to follow-up at each exit and six week survey. On average, 13.8 patients were enrolled for each of 31 clinicians participating in Cohorts 1 and 2 (range 8–17 patients). At baseline, study participants smoked a median of 10 cigarettes per day (IQR 5–20). Twenty-eight percent of participants reported considering quitting in the next month, 27% in the next six months, and 22% in the next year. Smoking was discussed in 64% of visits at baseline, and the average visit duration was 20.0 ± 9.2 minutes.

Exit surveys were completed an average of 1.7 days after the visit, with 24% completed more than 48 hours after the visit, and 2% completed after more than one week. On average, 6-week follow-up surveys were completed 42 days after the visit (range 36–71 days). Loss to follow up was minimal with only 6% (n = 50) of patients not completing the exit or 6 week survey. Less than 1% (n = 6) actively withdrew from the study after consent.

### Intervention assessment and feedback

As an initial assessment of the TMCP intervention, we asked clinician participants to rate characteristics of the training session (e.g. clarity of the concepts, usefulness of the workbook). Overall, participants reported that the supporting materials were helpful, the concepts clear, the utility of the sessions helpful, and the pacing of instruction appropriate. The skill practices with the standardized patients were rated as the most valuable training component by participants.

Clinician participants also completed surveys to assess self-reported levels of confidence and skills for addressing smoking cessation with their patients. This 7-item survey was administered before the intervention training sessions and again after the third session. Compared to baseline self-assessments, participants reported that their approach to smoking cessation was more effective, that it more effectively engaged patients to share their thoughts on quitting, and that it helped maintain a more positive relationship with patients.

## Discussion

Our goal was to develop and implement a new intervention which instructs clinicians to use the Teachable Moments Communication Process (TMCP). Grounded in previous direct observation research and the opportunities and challenges of the primary care setting, the TMCP uses brief and effective communication techniques to deliver a clear message about smoking cessation. Concurrently, the TMCP draws on behavioral counseling techniques to encourage the accurate assessment of the patient’s level of readiness, and enable an appropriate clinical response to that level of readiness [26-28]. The TMCP provides the clinician with a brief and tailored tool for delivering health behavior change advice that can be realistically translated into busy primary care practices.

The novelty of this intervention also lies in its use of multiple teaching modalities, including didactic presentation, video demonstration, and skill practices with standardized patients. This progressive format allows participants to develop their skills by building on each exercise. Feedback from self, peers and trainers provides continuous monitoring. Further, by measuring TMCP use in the primary care setting through direct observation, this approach bridges the gap between evidence-based strategies and implementation in the primary care setting [14]. Initial feedback from participants found the intervention to be useful, and self-reported assessments showed improved effectiveness in smoking cessation counseling.

We are actively collecting and analyzing data to evaluate the impact of the TMCP intervention on smoking cessation discussions and patient outcomes. If successful, the next steps will be to examine various pathways to widespread implementation, as well as the cost-effectiveness of and potential barriers to this implementation.

## Competing interests

The authors declare that they have no competing interests.

## Authors’ contributions

SF developed the concept for the study and the study design. She significantly contributed to the development and implementation of the intervention, interpretation of data analysis and drafting of the article. EA contributed to the development and implementation of the intervention, interpretation of data analysis and drafting of the article. MS contributed to the development and implementation of the intervention, interpretation of data analysis and drafting of the article. SM contributed to the development and implementation of the intervention and drafting of the article. TP contributed to the development and implementation of the intervention and drafting of the article. MJM contributed to the data analysis and interpretation of data and drafting of key sections of the article. All authors contributed to refining and revising the article for important intellectual content. All authors read and approved the final manuscript.

## Pre-publication history

The pre-publication history for this paper can be accessed here:

http://www.biomedcentral.com/1472-6963/12/109/prepub
